# Serum IL-21 levels associated with chronic hepatitis B and hepatitis B-related liver failure

**DOI:** 10.3892/etm.2014.1533

**Published:** 2014-02-10

**Authors:** HONG-MEI CHEN, HONG-LI LIU, YU-CONG YANG, XIAO-LI CHENG, YUE-FEI WANG, FAN-FAN XING, YING-REN ZHAO

**Affiliations:** Department of Infectious Diseases, First Affiliated Hospital of Medical College, Xi’an Jiaotong University, Xi’an, Shaanxi 710061, P.R. China

**Keywords:** interleukin-21, hepatitis B virus, clinical stages, peripheral blood mononuclear cells

## Abstract

The aim of the present study was to investigate the role of interleukin (IL)-21 in chronic hepatitis B virus (HBV) infection. IL-21 stimulates T and B cell responses and plays a role in the control of chronic viral infections. Serum IL-21 levels were measured by enzyme immunoassay in 109 patients with chronic HBV infection at various clinical stages, as well as in 19 healthy controls (HCs). The proportion of T cells producing IL-21 in the peripheral blood was assessed by intracellular cytokine staining and flow cytometry. Mean serum IL-21 levels in patients with chronic hepatitis B (CHB) and the HCs were 303.54±152.77 pg/ml and 68.24±9.06 pg/ml, respectively (P=0.003). In addition, the mean serum IL-21 level in patients with hepatitis B-related acute-on-chronic liver failure (HB-ACLF) was 455.38±412.38 pg/ml, which exhibited a statistically significant difference when compared with the HCs (P=0.000). Serum IL-21 levels were highest in the patients with HB-ACLF (455.38±412.38 pg/ml) and exhibited a significant difference when compared with the CHB patients (P=0.04). The mean serum IL-21 levels in patients with cirrhosis also increased, but there was no statistically significant difference when compared with the HCs (P=0.82). The frequency of IL-21+CD4+ cells also increased compared with the HCs and correlated with the number and percentage of lymphocytes in the peripheral blood. Serum IL-21 levels increased in CHB and HB-ACLF patients. Relatively low serum IL-21 levels in CHB may have a causal role in the persistence of HBV infection. Higher serum levels in HB-ACLF may activate T and B cells to eliminate the virus or injure the liver via the release of inflammatory cytokines.

## Introduction

Chronic hepatitis B virus (HBV) infection is a worldwide health problem with >400 million people infected (1,2). Patients with persistent infection of HBV, including those who are hepatitis B surface antigen (HBsAg)-positive without diagnosis of hepatitis, may progress to chronic hepatitis B (CHB), hepatitis B-related acute-on-chronic liver failure (HB-ACLF), cirrhosis and hepatocellular carcinoma (HCC). Disease progression results from the struggle between virus and host (3,4). The existence of different clinical stages is likely to be due to the various immune states that are possible with this infection (5,6). Cytokines are key molecules in the complex signaling network of humoral and cell-mediated immunity (4). Analyses of the changes in cytokine expression patterns during progression of the various clinical stages of chronic HBV infection may facilitate the understanding of pathogenesis.

Human interleukin (IL)-21 is a member of the type I cytokine family that is encoded by a gene on chromosome 4 (7). The mature form of human IL-21 contains 131 amino acids. The cytokine is produced by activated natural killer (NK) T cells and multiple CD4+ T cell subsets, including effector memory and central memory CD4+ T cells and differentiated T helper cell subsets polarized towards Th17 cell and T follicular helper phenotypes (8–10). IL-21 has important protective roles in the regulation of hematopoiesis, innate and adaptive immune responses and the regulation of autoimmunity (11–15). Similarly to other cytokines that signal through the common γ-chain subunit, IL-21 activates the Janus kinase (JAK)-family protein tyrosine kinases, JAK1 and JAK3, with JAK1 binding to the IL-21 receptor (IL-21R) and JAK3 binding to the common γ-chain (16–18). IL-21R-driven signaling results in the activation of signal transducer and activator of transcription molecules. IL-21R is expressed on a variety of immune cells, including T, B, NK and dendritic cells (DCs), as well as on non-immune cells such as fibroblasts, epithelial and endothelial cells (19). IL-21 has been shown to overcome virus-induced CD8+ T cell exhaustion, stimulate memory CD8+ T cells, stimulate the maturation and lytic ability of NK cells, suppress T regulatory cells and their production of IL-10 and stimulate precursor Th17 cells that have the ability to evolve into Th1 cells (20–22). It is also possible that DCs may become resistant to the suppressive effects of IL-21. Therefore, as an important inflammatory factor (23), IL-21 may be involved in liver injury via regulating the function of innate and adaptive immune competent cells and/or affecting the expression of other inflammatory cytokines. Previous studies have identified that IL-21 has an important role in murine lymphocytic choriomeningitis virus and human immunodeficiency virus infections (24–30). However, the role of IL-21 in chronic HBV infection remains unclear.

Therefore, the aim of the present study was to analyze serum IL-21 levels at various clinical stages of chronic HBV infection and determine whether IL-21 is associated with the progression of chronic HBV infection.

## Materials and methods

### Study subjects

A total of 109 patients with chronic HBV infection, admitted to the First Affiliated Hospital of Xi’an Jiaotong University (Xi’an, China) between January 2011 and October 2012, and 19 healthy controls (HCs) were enrolled in this study. All patients were HBsAg-positive for >6 months and had not received any antiviral treatment. The patients were divided into four groups. Firstly, the CHB group (n=35) included 24 hepatitis B e antigen (HBeAg)-positive and 11 HBeAg-negative cases. Patients were included in this group if they had serum alanine aminotransferase levels of >40 IU/l (upper normal limit, 40 IU/l), total bilirubin (TB) levels of <171 μM and plasma prothrombin activity (PTA) of >40%. Secondly, the HB-ACLF group (n=34) included 9 Child-Pugh B and 25 Child-Pugh C cases. The diagnosis of HB-ACLF was based on the clinical observation of grade ≥2 hepatic encephalopathy or a rapid increase in ascites that occurred within 4 weeks of the first signs of jaundice or coagulopathy. These clinical criteria were associated with the recent development of severe jaundice (TB levels, >171 μM) or rapidly rising levels of TB (>17.1 μmol/day) and a PTA of <40%. Thirdly, the clinical cirrhosis group (n=40), included 12 Child-Pugh A, 20 Child-Pugh B and 8 Child-Pugh C cases. Inclusion criteria consisted of the presence of long-term cirrhosis-associated complications, including ascites (with or without spontaneous bacterial peritonitis), varices and encephalopathy, and/or ultrasonographic observation of a small-sized liver with or without ascites and splenomegaly. Patients with the following concomitant conditions were excluded from the study: Hepatitis C (HCV) and D (HDV) infection, Wilson’s disease, autoimmune hepatitis, primary biliary cirrhosis and significant intake of alcohol (females, 20 g per day; males, 30 g per day). Finally, the HC group was recruited from students and staff at the First Affiliated Hospital of Xi’an Jiaotong University. The baseline characteristics of the patients are summarized in Table I.

### Ethical considerations

The study was conducted according to the Declaration of Helsinki and was approved by the Ethical Committee of the First Affiliated Hospital of Xi’an Jiaotong University. Written informed consent was obtained from all participants.

### Antibodies and other reagents

IL-21-Alex Fluor 647 and CD4-fluorescein isothiocyanate (FITC) fluorochrome conjugated antibodies with their isotype controls were purchased from BD Biosciences (San Jose, CA, USA).

### Serological and routine blood assays

The presence of HBsAg, HBeAg, anti-HBs, anti-HBc, anti-HBe, anti-HCV and anti-HDV was determined using commercial kits according to the manufacturer’s instructions (Quantification kit; Abbott Laboratories, Green Oaks, IL, USA).

### Serum cytokine concentration

Serum concentrations of IL-21 were measured in duplicate using a commercial human IL-21 platinum enzyme-linked immunosorbent assay kit (eBioscience, Inc., San Diego, CA, USA), according to the manufacturer’s instructions. The lower detection limit of the kit was 20 pg/ml.

### Intracellular cytokine staining (ICS)

All cells used in the study were cultured at 37°C in a humidified atmosphere containing 5% CO_2_ (31). Heparinized whole blood (200 μl) was stimulated with 20 ng/ml phorbol-12-myristate-13-acetate (PMA; Sigma-Aldrich, St. Louis, MO, USA) and 1 μg/ml calcium ionomycin (Sigma-Aldrich) in the presence of monensin (3 μM; Sigma-Aldrich) for 5 h. The control consisted of unstimulated cells under identical conditions. Next, 1 ml 1X BD FACS Lysing Solution (BD Biosciences; diluted with 10X solution 1:10 with deionized water prior to use) was added to the activated and unstimulated whole blood samples, mixed gently and incubated for 10 min at room temperature. Wash buffer (2 ml) was added to each tube and centrifuged at 500 × g for 5 min at room temperature. The cells were harvested and stained with CD4-FITC for 30 min at room temperature in the dark and then washed and fixed with 100 μl Medium A (Caltag Fix&Perm™ reagent; Invitrogen Life Technologies, Carlsbad, CA, USA) for 15 min. Following washing and permeabilization for 25 min with 100 μl Medium B (Invitrogen Life Technologies), the cells were incubated at room temperature for 30 min in the dark with IL-21-Alex Fluor 647 or the isotype controls. Wash buffer (2 ml) was added to each tube and centrifuged at 500 × g for 5 min at room temperature. The supernatant was decanted and 200 μl paraformaldehyde (1%) in phosphate-buffered saline was added. The pellet was resuspended and stored at 4°C in the dark prior to flow cytometry analysis. Samples were analyzed within 24 h (32–34).

### Statistical analysis

All statistical analyses were performed using SPSS software version 17.0 (SPSS, Inc., Chicago, IL, USA). Data are expressed as the mean ± SD. Parametric data were analyzed using one-way analysis of variance. For the homogeneity test of variance, Levene’s test was used. Dunnett’s T3 test was used in case of heterogeneity of variance among multiple groups. A corrected P-value was calculated by Bonferroni correction for multiple comparisons when the uncorrected P-value was <0.05. Association between variables was evaluated using Spearman’s correlation coefficient. All statistical analyses were based on two-tailed hypothesis tests where P<0.05 was considered to indicate a statistically significant difference.

## Results

### Cross-sectional analysis of serum IL-21 levels

**Figure 1:**
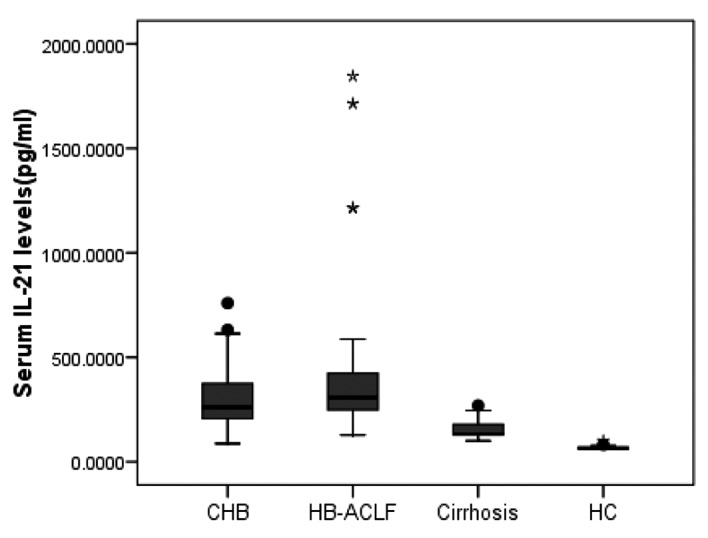
Comparison of serum IL-21 concentrations in the four groups. Horizontal bars indicate the median values in each group. P-values indicate the significance of interclass comparisons using one-way analysis of variance. Corrected P-values were calculated by Bonferroni correction for multiple comparisons when the uncorrected P-value was <0.05. P=0.04, CHB vs. HB-ACLF; P=0.033, CHB vs. cirrhosis; P=0.003, CHB vs. HC; P=0.000, HB-ACLF vs. cirrhosis; P=0.000, HB-ACLF vs. HC ; P=0.82, cirrhosis vs. HC. CHB, chronic hepatitis B; HB-ACLF, hepatitis B-related acute-on-chronic liver failure; HC, healthy controls; IL-21, interleukin-21.

Serum IL-21 concentrations in the CHB, HB-ACLF, cirrhosis and HC groups are shown in Table II. The mean serum level of IL-21 in the HB-ACLF group was 455.38±412.38 ng/ml, which was significantly higher than that of the CHB, cirrhosis and HC groups. The mean serum level of IL-21 in the CHB group was 303.54±152.77 ng/ml, which was higher compared with that of the cirrhosis and HC groups. The mean level of IL-21 in the cirrhosis group also increased, but there was no statistically significant difference when compared with the HC group (Fig. 1).

### Cross-sectional analysis of IL-21-secreting CD4+ T cells by ICS

**Figure 2:**
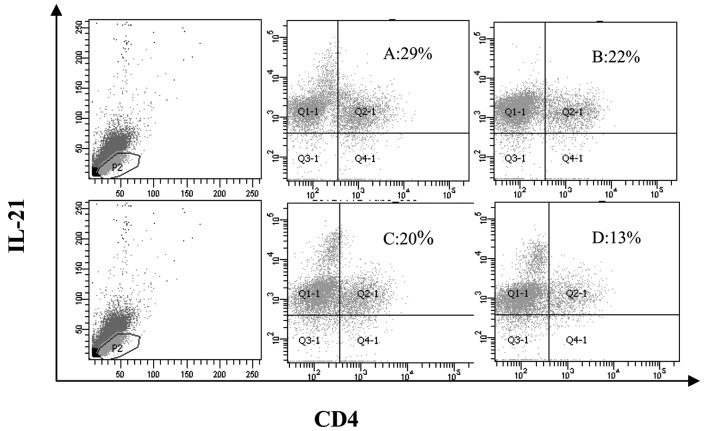
Analysis of IL-21-producing cells by ICS. The frequencies of IL-21-secreting CD4+ T cells in the CHB, HB-ACLF and cirrhosis groups are compared with the HC group. Gated on lymphocytes. A, CHB; B, HB-ACLF; C, cirrhosis; D, HC; ICS, intracellular cytokine staining; CHB, chronic hepatitis B; HB-ACLF, hepatitis B-related acute-on-chronic liver failure; HC, healthy controls; IL-21, interleukin-21.

**Figure 3:**
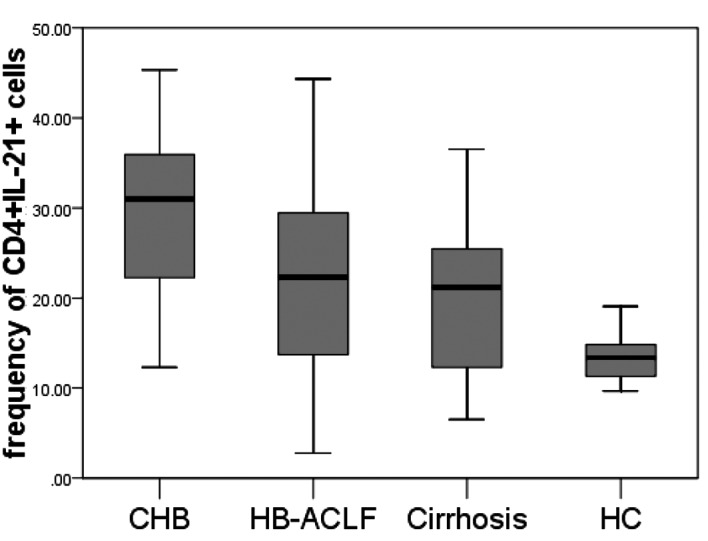
Comparison between the frequencies of CD4+IL-21+ cells in the CHB, HB-ACLF, cirrhosis and HC groups. P=0.003, CHB vs. HB-ACLF; P=0.000, CHB vs. cirrhosis; P=0.000, CHB vs. HC; P=0.88, HB-ACLF vs. cirrhosis; P=0.003, HB-ACLF vs. HC; P=0.04, cirrhosis vs. HC. CHB, chronic hepatitis B; HB-ACLF, hepatitis B-related acute-on-chronic liver failure; HC, healthy controls; IL-21, interleukin-21.

The frequencies of IL-21+CD4+ T cells in PMA/ionomycin-stimulated lymphocytes in the four groups are shown in Table II and Figs. 2 and 3. The frequency of IL-21+CD4+ T cells in the CHB, HB-ACLF and cirrhosis groups was significantly higher compared with the HC group (P<0.05). The mean percentage of IL-21+CD4+ T cells in the CHB group was 29.52±9.08%, which was the highest compared with the remaining groups. However, the percentage of IL-21+CD4+ T cells in the HB-ACLF group was similar to the cirrhosis group, but with no statistically significant difference.

### Association between IL-21 concentrations and lymphocytes in the peripheral blood

**Figure 4:**
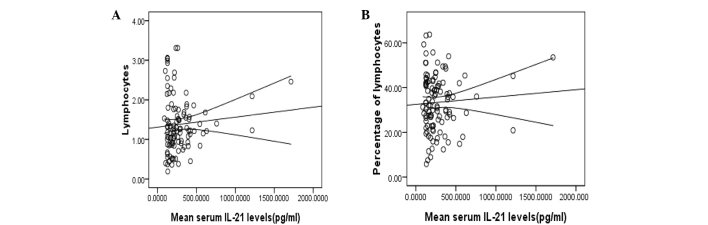
Correlation between serum IL-21 levels and (A) lymphocyte counts (r=0.081;P=0.402) and (B) percentages of lymphocytes (r=0.06; P=0.534). IL-21, interleukin-21.

There were no correlations between IL-21 concentrations and the frequency (r=0.081; P=0.402) or percentage of lymphocytes (r=0.06; P=0.534) in the peripheral blood (Fig. 4).

### Association between the frequency of IL-21+CD4+ cells and lymphocytes in the peripheral blood

**Figure 5:**
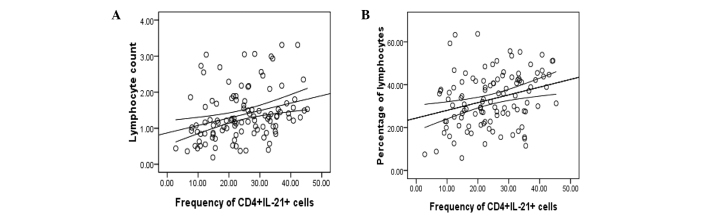
Frequency of IL-21+CD4+ cells positively associated with the (A) frequency of lymphocytes (r=0.296; P=0.002) and (B) percentage of lymphocytes (r=0.293; P=0.002) in the peripheral blood. IL-21, interleukin-21.

The frequency of IL-21+CD4+ cells positively correlated with the frequency (r=0.296; P=0.002) and percentage of lymphocytes (r=0.293; P=0.002) in the peripheral blood (Fig. 5).

## Discussion

The majority of viral infectious diseases are self-limiting, such as measles, mumps and rhinovirus (35). Only a few result in chronic infections, including HBV, HCV and HIV (36). The reason that certain viruses only cause acute infections, while others result in chronic disease has not been elucidated. The majority of liver damage caused by viral infections is mediated by the host immune response (6). Acquired and innate immunity are hypothesized to be involved in the pathogenesis of viral infectious diseases (3,5).

Patients infected with HBV may develop CHB, ACLF, cirrhosis and HCC. In the current study, serum IL-21 concentrations were measured at various clinical stages of chronic HBV infection and the results demonstrated that serum IL-21 levels were highest in the HB-ACLF and CHB groups.

At present, CHB pathogenesis remains unclear. HBV itself has no direct cytotoxic effects. Immune pathological injury to the liver may be induced by the virus, which maintains *in vivo* replication by activating monocyte/macrophage phagocytosis, processing and triggering an immune response. Cytokines secreted by immune cells are important in the occurrence, development and progression of CHB. Chronicity of HBV infection may be more frequent in T and B cell immunodeficiency (37). IL-21 stimulates T and B cell responses and is important for the control of chronic viral infections. Therefore, serum IL-21 levels in patients with CHB may be involved with the development of chronic HBV infection.

The results of the present study revealed that serum IL-21 levels were significantly elevated in the CHB group. However, the elevated levels in the CHB group were lower compared with previously detected levels in the acute stage of hemorrhagic fever with renal syndrome (data not shown). A previous study demonstrated that serum IL-21 levels in CHB patients treated with interferon (IFN) and nucleoside analogues increased as the viral loads decreased (38). There are two antiviral mechanisms of IFN activity. The first is direct antiviral action and the second is associated with immunoregulation (39). By contrast, nucleoside analogues interfere directly with the replication of the virus (40). However, a previous study has shown that certain nucleoside analogues have immunomodulatory effects (41). There is no general consensus on whether immune mechanisms are involved in nucleoside analogue efficacy. However, increased IL-21 levels have been associated with viral elimination (42). Relatively low serum IL-21 levels in CHB may play a causal role in the persistence of HBV infection.

Since acute HBV infection commonly begins with no significant symptoms, the current study did not include cases of acute HBV infection. Thus, dynamic changes in serum IL-21 levels in acute HBV infection require further study.

ACLF, an acute hepatic insult that frequently presents with jaundice and coagulopathy, is complicated within 4 weeks by ascites and/or encephalopathy in patients previously diagnosed or undiagnosed with chronic liver disease (43). In China, >80% of ACLF cases are infected with HBV and the relapse of hepatitis occasionally results in liver failure (44). The pathogenic mechanisms of HB-ACLF remain largely unknown. Genetic mutations of the virus and derangements in the hosts themselves can result in hepatic injury and malfunction of the liver due to immunological damage, ischemia and anoxia or endotoxemia, which may occur sequentially or simultaneously with the virological rebound (45). A number of studies have focused on immune damage. Monocytes, DCs, NK cells, CD4+CD25+ regulatory T cells, Th17 cells and other immunologically competent cells have been shown to be involved in the pathogenesis of HB-ACLF (46–49).

The results of the present study demonstrated that serum IL-21 levels were elevated in patients with HB-ACLF, which is consistent with the results of Hu *et al* (50). Serum IL-21 levels in the HB-ACLF group were higher than those in the CHB group. Immunological damage is the main factor of liver injury in HB-ACLF. Higher serum IL-21 levels in HB-ACLF may play a positive or negative role, since IL-21 may activate T and B cells to eliminate the virus or injure the liver by the release of inflammatory cytokines.

Patients with cirrhosis caused by HBV infection often have a variety of complications, including chronic liver failure and HCC. IL-21 levels were measured in patients with CHB, HB-ACLF and cirrhosis over time. The results showed that serum IL-21 levels in the CHB and HB-ACLF groups were higher than in patients with cirrhosis caused by HBV infection. CD4+IL-21+ T cell frequency in the CHB group was higher compared with the cirrhosis group and correlated with the peripheral blood lymphocyte counts. While the mechanism by which these observations occurred has not yet been determined, one possible contributing factor is the decrease in the numbers of peripheral blood and lymphocytes due to the hypersplenism that accompanies portal hypertension in cirrhosis.

In conclusion, administration of IL-21 is well-tolerated in humans when compared with other cytokines. IL-21 is currently being evaluated in clinical trials as an immunotherapy agent against melanoma and renal cell carcinoma (51). The results of the present study indicate that IL-21 should also be considered as an immunotherapeutic tool for HBV-infected individuals.

## Figures and Tables

**Table I:** Clinical characteristics of the study subjects.

	Groups			
	
Characteristics	CHB	ACLF	Cirrhosis	HC
Patients, n	35	34	40	19
Age, years	38.53±15.63	37.44±12.40	49.88±12.90	21.93±0.83
Gender, male/female, n	29/6	26/8	31/9	7/12
ALT, IU/l (normal scale, 0–40)	106.47±127.50	104.27±114.37	56.48±53.29	NA
TBIL, μM (normal scale, 0–17.1)	46.08±35.12	306.38±206.50	73.44±71.25	NA
WBC, ×10^9^	4.89±2.06	4.24±1.57	3.83±2.21	NA
Lymphocyte count, ×10^9^	1.55±0.65	1.29±0.48	1.29±0.90	NA
Percentage of lymphocytes, %	33.58±10.66	32.22±12.24	33.90±14.57	NA
HBV DNA, log_10_ IU/ml	5.56±1.38	3.79±2.38	3.79±2.75	NA

CHB, chronic hepatitis B; ACLF, acute-on-chronic liver failure; HC, healthy controls; NA, not available; ALT, alanine aminotransferase; TBIL, total bilirubin; WBC, white blood cell; HBV, hepatitis B virus.

**Table II:** Mean serum IL-21 concentrations and IL-21+CD4+ cell frequencies in the study groups.

Groups	Serum IL-21 concentrations, pg/ml	Frequency of IL-21+CD4+ cells, %
CHB	303.54±152.77	29.52±9.08
HB-ACLF	455.38±412.38	22.32±10.66
Cirrhosis	154.38±39.83	20.49±8.62
HC	68.24±9.06	13.61±2.87

CHB, chronic hepatitis B; HB-ACLF, hepatitis B-related acute-on-chronic liver failure; HC, healthy controls; IL-21, interleukin-21.
